# Diagnostic accuracy of conventional oral examination for detecting oral cavity cancer and potentially malignant disorders in patients with clinically evident oral lesions: Systematic review and meta‐analysis

**DOI:** 10.1002/hed.26992

**Published:** 2022-01-29

**Authors:** Munira Essat, Katy Cooper, Alice Bessey, Mark Clowes, James B. Chilcott, Keith D. Hunter

**Affiliations:** ^1^ ScHARR The University of Sheffield Sheffield UK; ^2^ School of Clinical Dentistry The University of Sheffield Sheffield UK

**Keywords:** biopsy, conventional oral examination, diagnostic accuracy, oral cancer, systematic review

## Abstract

This systematic review evaluates the diagnostic accuracy of conventional oral examination (COE) versus incisional or excisional biopsy for the diagnosis of malignant and/or dysplastic lesions in patients with clinically evident lesions. Searches were conducted across five electronic databases from inception to January 2020. Meta‐analyses were undertaken, where appropriate. Among 18 included studies, 14 studies were included in the meta‐analysis, giving summary estimates for COE of 71% sensitivity and 85% specificity for the diagnosis of dysplastic and/or malignant lesions. The pooled diagnostic accuracy of identifying malignant‐only lesions was reported in seven studies, giving a pooled estimate of 88% sensitivity and 81% specificity. Diagnostic accuracy of different types of dental/medical professionals in identifying dysplastic or malignant lesions gave varying estimates of sensitivity and specificity across three studies. Further research is needed to improve the diagnostic accuracy of COE for early detection of dysplastic and malignant oral lesions.

**Abbreviations**
CI: confidence interval
COE: conventional oral examination
OC: oral cancer
OCC: oral cavity cancer
PRISMA: Preferred Reporting Items for Systematic Reviews and Meta‐Analyses
QUADAS‐2: Quality Assessment of Diagnostic Accuracy Studies‐2
ROC: receiver operating characteristic curve

## 1 INTRODUCTION

Oral cancer (OC), defined as cancers of the lips, tongue, cheeks, floor of the mouth, hard and soft palate, sinuses, and pharynx, has an incidence of more than 300 000 cases per year.^1^, ^2^, ^3^ The prognosis of OC is improved when it is detected at an early stage, with a 5 year survival rate of 75% for stage I disease which is drastically reduced to 30% at stage IV of the disease.^2^, ^4^ This review focusses on cancer of the oral cavity, including the lips, the lining inside the cheeks and lips, the front two thirds of the tongue, the upper and lower gums, the floor of the mouth under the tongue, the bony roof of the mouth, and the small area behind the wisdom teeth.

The diagnostic pathway for identifying oral cavity cancers (OCC) and oral potentially malignant disorders (OPMD) in patients with a clinically evident lesion starts with a full clinical history, followed by conventional oral examination (COE), which includes a thorough head and neck examination, evaluation of oral mucosa by visual inspection under incandescent overhead light or halogen illumination available on the dental chair, and palpation.^5^, ^6^, ^7^ Features of COE which may be indicative of OCC or oral dysplastic lesions include non‐homogenous appearance such as changes in surface texture, color and size; loss of surface integrity; alteration in the surface, for example, slightly raised lesions; nonhealing ulceration or tethering of the tissues (suggesting deeper invasion).^5^, ^8^ The suspicious lesion then undergoes histological examination using either incisional or excisional biopsy, the gold standard for diagnosing cancer or epithelial dysplasia. However, this diagnostic pathway approach has limitations in that the findings of COE are subjective and dependent on the experience and expertise of the clinician, while biopsy is invasive and can lead to morbidity. Although various aids and adjuncts to COE have been developed,^9^, ^10^, ^11^ there is little consensus on which, if any, are most reliable, and standard care in many countries remains COE followed by biopsy, if needed.

This systematic review evaluates the diagnostic accuracy of COE (visual inspection) compared with incisional or excisional biopsy (gold standard) for the diagnosis of OCC and/or OPMDs in patients with a clinically evident oral lesion.

## 2 METHODS

A systematic review was undertaken in accordance with the general principles recommended by expert consensus guidelines for the conduct of diagnostic accuracy systematic reviews^12^, ^13^ and the Preferred Reporting Items for Systematic Reviews and Meta‐Analyses (PRISMA) statement.^14^ As this study met criteria for nonhuman subject research, approval by the University of Sheffield Research Ethics Committee was not required.

### 2.1 Data sources and searches

A comprehensive literature search was undertaken to identify potentially relevant studies. Searches were conducted in several databases including MEDLINE, EMBASE, CINAHL, and the Cochrane Library (including the CENTRAL Register of Controlled Trials and Cochrane Database of Systematic Reviews) from inception to January 2020. The search strategy used free text and thesaurus terms and combined synonyms relating to the condition (e.g., oral cancer, oral lesion, premalignant) with diagnostic testing terms (including a high‐precision filter developed by McMaster University). No date or language restrictions were applied. Searches were supplemented by examination of the reference lists of relevant studies including existing systematic reviews and contact with key experts in the field. Further details of the search strategy are provided in Appendix S1.

### 2.2 Study selection

All titles and abstracts were examined for inclusion by one reviewer. Any citations that clearly did not meet the inclusion criteria (e.g., nonhuman or unrelated to oral lesions) were excluded. A check of inclusion decisions was performed by a second reviewer for 10% of titles and abstracts with a very good agreement (Kappa = 0.81). All full text articles were then examined independently by two reviewers. Any disagreements in the selection process were resolved through discussion. In order to maintain relevance to current diagnostic approaches only studies published from January 1990 and studies from developed countries with comparable health system were included. Details of the selection criteria are provided in Appendix S2.

### 2.3 Data extraction

Data relating to study design, patient characteristics, diagnostic accuracy, and outcomes were extracted by one reviewer into a standardized data extraction form and independently checked for accuracy by a second reviewer. Any discrepancies were resolved through discussion to achieve agreement. Where multiple publications of the same study were identified, data were extracted and reported as a single study. For papers focusing primarily on adjunctive tests to clinical examination, subgroup results relating to COE only were extracted. If diagnostic accuracy of different dental and medical professionals performing the index test (COE) were evaluated within a single study, results were extracted separately for each professional group.

### 2.4 Clinical outcomes assessed

Diagnostic outcomes were extracted for two sets of data (where reported): (1) dysplastic lesions and malignant lesions together, and (2) malignant lesions alone. All the studies in this review included patients with clinically evident lesions, hence a negative test (negative lesion) refers to a lesion that has been detected but it is determined to be neither dysplastic nor malignant based on the clinical features. The authors' cut‐off for positive/negative result for OCC and/or epithelial dysplasia was accepted, and the algorithm used to generate the cut‐off was noted. For (1) that is, assessment of dysplastic and malignant lesions taken together, COE findings were considered positive where there was any level of concern for dysplasia or cancer, including atypical, abnormal, mild/moderate/severe dysplasia, carcinoma‐in‐situ, invasive cancer, or any other result that implied the presence of dysplasia/malignancy. Conversely, results such as inflammation or no dysplasia were considered negative, along with benign and normal results. Gold standard (incisional or excisional biopsy) results positive for cancer or any grade of dysplasia were also considered positive. For (2) that is, assessment of malignant lesions alone, results (for both COE and incision/excision) were considered positive if they indicated cancer/carcinoma, but negative if they indicated dysplasia or carcinoma‐in‐situ.

### 2.5 Quality assessment

The methodological quality of each included study were assessed using the Quality Assessment of Diagnostic Accuracy Studies‐2 (QUADAS‐2) tool^15^ across the following key domains: patient selection (a consecutive or random sample of patients, avoidance of case–control study design and avoidance of inappropriate exclusions), index test (COE, interpreted without knowledge of reference standard and if prespecified threshold used), reference standard (validity of the reference standard and blinding of the pathologist to COE), flow and timing (time span between COE and histopathology, all patients received same reference standard, and missing data). Each domain was assessed in terms of risk of bias and the concern regarding applicability to the review (the latter for the first three domains only). The sub‐domains for each domain include a number of signaling questions to guide the overall judgment about whether a study is at high, low, or an unclear risk of bias. The studies were assessed by one reviewer and independently checked by another reviewer.

### 2.6 Data synthesis and analysis

Data were tabulated and discussed in a narrative synthesis. Meta‐analyses were undertaken, where appropriate to estimate a summary measure of effect on relevant outcomes using the random effects model to allow for inter‐study variability. Statistical analyses were performed using MetaDTA software, an interactive online application for conducting meta‐analysis of diagnostic test accuracy studies.^16^ Pooled estimates of sensitivity and specificity are presented as point estimates and 95% confidence intervals (CIs) for COE versus biopsy. Results were recalculated from raw data presented in publications if alternative metrics of diagnostic accuracy were originally reported. In cases where articles used different cut‐offs for defining positive and negative test/biopsy results, the data were synthesized narratively and not included in the meta‐analysis.

## 3 RESULTS

### 3.1 Study flow

**FIGURE 1:**
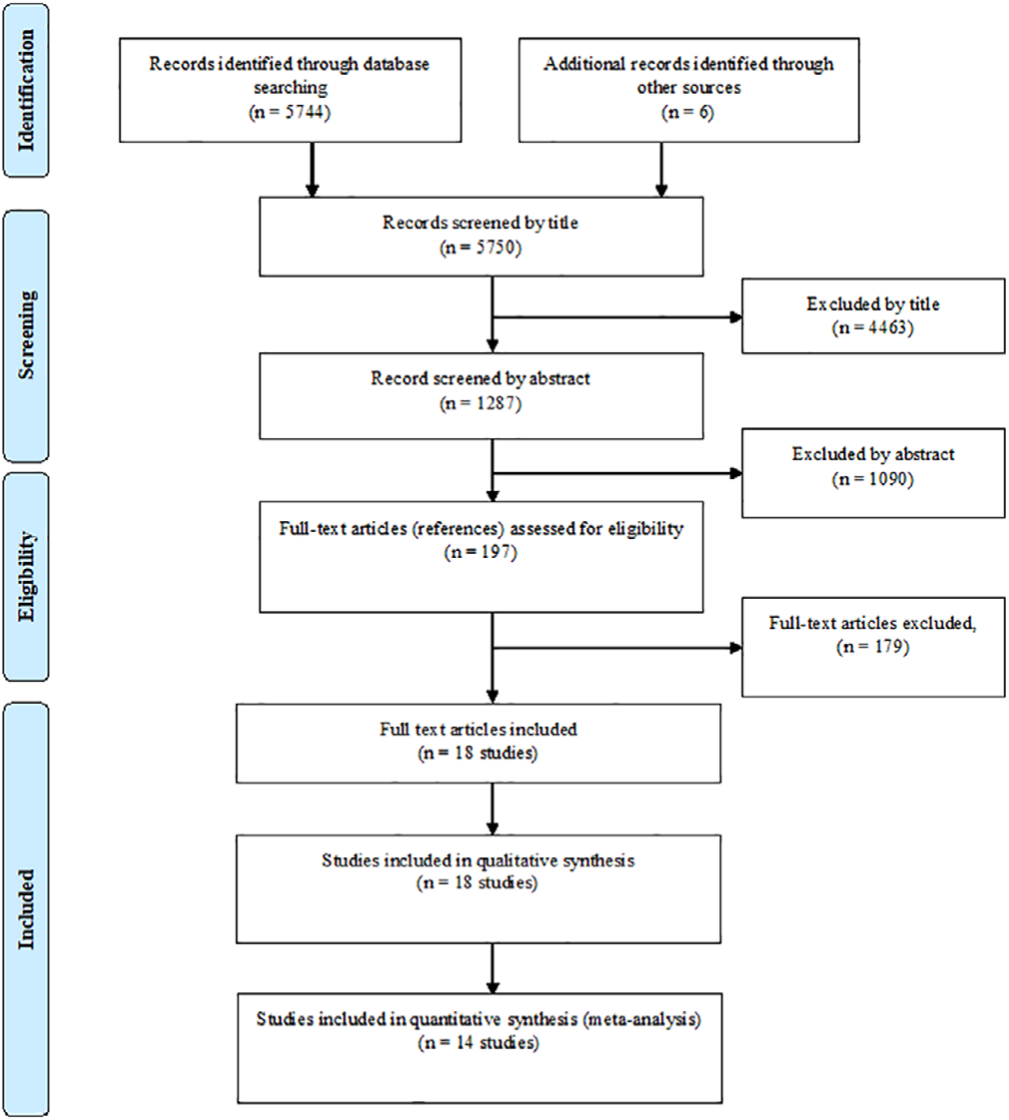
Study flowchart [Color figure can be viewed at wileyonlinelibrary.com]

Figure 1 summarizes the process of identifying and selecting relevant literature. Of the 5750 citations identified, 18 studies met the inclusion criteria (14 studies were eligible for inclusion in the meta‐analysis). The majority of excluded studies either did not present data on OCC and/or OPMDs, did not use COE as a standalone approach to identify OCC and/or OPMDs, provided insufficient outcome data, or were conducted in nonrelevant countries.

### 3.2 Study and patient characteristics

The design and patient characteristics of the 18 included studies are summarized in Table 1. All included studies investigated the diagnostic accuracy of COE compared with incisional biopsy apart from one study which compared COE versus excision of full lesions^17^ and another study^18^ which compared COE versus histologic confirmation without any further details. The studies were published between 1998^19^ and 2016^17^ and various methods were used for performing biopsy, including punch (*n* = 1),^20^ scalpel (*n* = 5),^21^, ^22^, ^23^, ^24^, ^25^ punch or scalpel (*n* = 1),^26^ punch or wedge (*n* = 1),^27^ surgical unspecified (*n* = 2),^19^, ^28^ scalpel or surgical (*n* = 1),^29^ and in six studies the method used for biopsy was not reported.^18^, ^30^, ^31^, ^32^, ^33^, ^34^ The size of the studies varied considerably with the number of participants ranging from 10^19^ to 3067^33^ and the number of lesions ranging from 28^19^ to 3127.^33^ The proportion of male patients ranged from 33%^28^ to 75%^27^ and the mean age ranged from 45^25^, ^30^ to 65 years.^17^ The comparability of study populations in terms of previous history of cancer was difficult to determine as this information was infrequently reported. Four studies^27^, ^30^, ^32^, ^34^ included some patients with a history of cancer, three studies^17^, ^22^, ^26^ stated exclusion of participants with previous history of cancer and the remainder of the studies did not report this data. The studies were mainly conducted in either secondary or tertiary care settings apart from Brocklehurst et al.^18^ and Patel et al.^33^ in which the study assessed the diagnostic test accuracy of different members of the dental team in different care setting. The index test (COE) was performed by various dental or medical professionals including oral surgeons,^21^ neck and head surgeons or dental oncologists,^19^ oral medicine specialists,^22^ otolaryngologists,^20^ oral and maxillofacial surgeons^28^ dental oncology specialists^23^, ^32^ primary care dentists,^18^, ^33^ dental hygienists and dental hygiene therapists.^18^

**TABLE 1:** COE versus biopsy or excision: Study characteristics

Author, year country study design	Population	History of oral cancer/lesions	Sample size (no. of lesions)	Mean age (years)	No. of males (%)	Index test	Reference standard	Dental professional performing index test	Prevalence: dysplasia or malignant lesions	Prevalence: malignant lesions
Allegra 2009^20^ Italy NR	Oral mucosa lesions	NR	32 (45)	59	19/32 (59%)	COE	Punch biopsy	Experienced otolaryngologist	30/45 (67%)	7/45 (16%)
Bhoopathi 2011^21^ USA Retrospective^a^	Atypical lesions or positive by brush biopsy	NR	148 (148)	55	80/148 (54%)	COE	Scalpel biopsy	Oral surgeons	12/148 (8%)	NR
Brocklehurst 2015^18^ UK Retrospective	Standardized clinical photographs of mouth cancer, PMDs and benign lesions of the oral mucosa	NR	90 (90)	NR	NR	COE	Histological confirmation	Primary care dentists (*N* = 96)	35/90 (39%)	NR
								Hygienists/therapists (*N* = 63)	35/90 (39%)	NR
								Hospital based dentists (*N* = 9)	35/90 (39%)	NR
								Dental nurses (*N* = 24)	35/90 (39%)	NR
Chainani‐Wu 2015^26^ USA Cross‐sectional, consecutive	Oral LP, ELP, or EP on clinical examination with no current history of oral cancer	No	43 (77)	61	23/43 (54%)	COE	Punch or scalpel biopsy	NR	17/77 (22%)^b^	6/77 (8%)
Epstein 2003^27^ USA Prospective	Treated within past 2 years for upper aerodigestive tract or pulmonary carcinoma but no current treatment for oral cancer	Y (history of cancer)	81 (96)	61	61/81 (75%)	COE	Punch or wedge biopsy	NR	30/96 (31%)^c^	NR
Farah 2012^22^ Australia Prospective	Clinically suspicious oral mucosal white or mixed red/white lesion; no known oral epithelial dysplasia or SCC	No	112 (118)	59	46/112 (41%)	COE	Scalpel biopsy	Oral medicine specialists	28/118 (24%)	NR
Forman 2015^30^ USA Retrospective	Oral lesion with biopsy and pathology report with unequivocal clinical impression and histologic diagnosis	Y: 12.9% had a history of cancer	1003 (1003)	45	491/1003 (49%)	COE	Biopsy	Surgeons (25–30 years experience) or residents (< 5 years experience)	74/1003 (7.4%)	NR
Gillenwater 1998^19^ USA NR	Known or suspected premalignant or malignant oral cavity lesions and normal tissue from same patients	NR	10 (28)	NR	NR	COE	Surgical biopsy	Experienced neck and head surgeon or dental oncologist	17/28 (61%)	NR
Hanken 2013^28^ Germany Prospective	Suspicious oral premalignant lesions but with no current advanced SCC	NR	60 (60)	NR (range 38–82)	20/60 (33%)	COE	Surgical biopsy	Experienced oral and maxillofacial surgeon	54/60 (90%)	3% (2/60)
Jayaprakash 2013 (abst)^31^ USA NR	Potentially malignant white or white‐red oral mucosal lesions	NR	146 (255)	NR	NR	COE	Biopsy	NR	184/255 (72%)	NR
Jayaprakash 2009^32^ USA NR	Clinically suspicious oral lesions or recently diagnosed untreated premalignant lesions or cancer, history of previously treated oral cancer but no evidence of cancer recurrence and no active malignancy treatment	Y: 47% previous head and neck cancer	60 (249)	60	41/60 (68%)	COE	Biopsy	Specialist dental oncologist	170/249 (68%)	15/249 (6%)^d^
Kammerer 2015^29^ Germany Prospective	Potentially malignant oral disorders	NR	44 (50)	60	25/44 (57%)	COE	Scalpel/surgical biopsy	NR	10/50 (20%)	7/50 (14%)
Koch 2011^23^ Germany NR	Clinically suspicious epithelial lesions or diagnosed oral mucosal lesion as SCC	NR	78 (78)	62	46/78 (59%)	COE	Scalpel biopsy	Specialist dental oncologist	33/78 (42%)	30/78 (38%)
Marzouki 2012^34^ Canada Prospective	Strong history of smoking, alcohol and suspicious lesion referred by GP, or previous history of cancer but cancer free and having regular follow‐ups	Y: 68% previous head and neck cancer	33 (33)	62	49/85 (58%)	COE	Biopsy	Head and neck oncology staff person	13/33 (40%)	NR
McIntosh 2009^24^ ^,^ ^e^ Australia NR	Clinically suspicious oral mucosal white lesion	NR	50 (50)	57	23/50 (46%)	COE	Scalpel biopsy	NR	9/50 (18%)	NR
McNamara 2012^25^ USA NR	Undergoing initial oral evaluation and routine dental care	NR	NR (34)	45	67/130 (52%)	COE	Scalpel biopsy	Resident, oral and maxillofacial pathology	3/34 (9%)	NR
Patel 2011^33^ New Zealand Retrospective	All lesions involving soft tissues of mouth: tongue, gingiva, unattached mucosa, and the lips to the vermillion‐skin junction	NR	3067 (3127)	49	1308/3067 (43%)	COE	Biopsy	All clinicians combined	391/2517 (16%)	66/2517 (2.6%)
								General dental practitioner	32/404 (8%)	3/404 (0.7%)
								Specialist dentist with postgraduate qualifications	350/2079 (17%)	58/2079 (2.8%)
Piazza 2016^17^ Italy Prospective	Not treated for OC/OP with LPs and EPs and not been biopsied	No	128 (128)	65	54/128 (42%)	COE	Excisional biopsy^f^	NR	87/128 (68%)	NR

Abbreviations: Abst, conference abstract; CIS, carcinoma in situ; COE, conventional oral examination; ELP, erythroleukoplakia; EPs, erythroplakias; LPs, leukoplakias; NR, not reported; OC, oral cavity; OP, oropharyngeal; PMDs, premalignant diseases; SCC, squamous cell carcinoma.

^a^ Consecutive cohort but retrospective analyses.

^b^ For severe dysplasia and cancer only.

^c^ For cancer or CIS only, not dysplasia.

^d^ Calculated for invasive SCC and other carcinomas (one salivary gland carcinoma and two verrucous carcinomas with SSC component).

^e^ Data reported for Microlux/DL examination but was same as clinical provisional diagnosis.

^f^ Excisional biopsy of the entire lesion under local or general anesthesia regardless of the appearance at COE.

The classification and cut‐off used to define positive/negative tests for dysplastic or malignant lesions in the included studies was broadly similar apart from two studies^26^, ^27^ which were excluded from the meta‐analysis. Chainani‐Wu et al.^26^ categorized a positive test as severe dysplasia, carcinoma in situ or carcinoma, while a negative test included mild or moderate dysplasia and hyperkeratosis. Epstein et al.^27^ considered carcinoma or carcinoma in situ as a positive test and dysplasia, keratosis, hyperkeratosis, and hyperplasia as negative test. The majority of the included studies defined a positive result as a lesion with any degree of dysplasia or malignancy, while a negative result was defined as having no dysplasia or being benign. Further details of definitions used by each of the included studies are described in Table 3 for dysplastic or malignant lesions and in Table 4 for malignant lesions. Prevalence of dysplastic or malignant lesions varied widely in the 18 included studies, ranging from 7.4%^30^ to 90%.^28^ The prevalence of malignant‐only lesions was reported in seven studies and ranged from 0.7%^33^ to 38%.^23^

### 3.3 Quality assessment

The overall methodological quality of the included studies is summarized in Table 2. Generally, the ratings indicated either low or unclear risk of bias in the majority of studies. The domain with the highest risk of bias concerned “flow and timing” in four studies,^19^, ^21^, ^26^, ^27^ primarily due to the lack of inclusion of all patients in the analyses, for example, participants missing or excluded from analysis and no explanation given. This was followed by patient selection which was rated as high risk of bias in two studies.^21^, ^27^ Bhoopathi et al.^21^ only included patients with lesions that were atypical or positive by brush biopsy and Epstein et al.^27^ included patients who were treated within past 2 years for upper aerodigestive tract or pulmonary carcinoma. None of the studies specified the time interval between the index test (COE) and the reference standard (biopsy) and were therefore judged as unclear on this aspect. Furthermore, nine studies^18^, ^21^, ^23^, ^25^, ^28^, ^30^, ^31^, ^33^, ^34^ did not report on whether the pathologist was blinded to the index test when interpreting the histopathologic findings of the biopsy. However, in all the studies the index test (COE) was interpreted without the knowledge of the reference standard. With respect to applicability, all the studies except two^21^, ^27^ had a low risk of bias. These two studies^21^, ^27^ only included selected patients.

**TABLE 2:** Risk of bias summary: Judgments of risk of bias for each included study

Study	Risk of bias				Applicability concerns		
	Patient selection	Index test	Reference standard	Flow and timing	Patient selection	Index test	Reference standard
Allegra 2009^20^	?	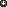	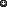	?	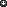	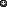	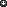
Bhoopathi 2011^21^	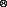	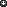	?	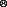	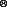	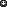	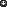
Brocklehurst 2015^18^	?	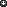	?	?	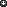	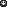	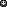
Chainani‐Wu 2015^26^	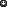	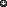	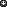	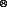	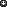	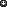	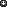
Epstein 2003^27^	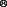	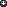	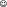	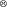	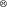	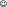	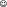
Farah 2012^22^	?	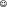	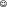	?	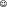	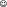	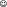
Forman 2015^30^	?	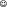	?	?	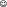	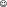	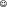
Gillenwater 1998^19^	?	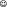	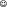	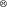	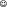	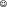	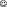
Hanken 2013^28^	?	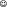	?	?	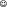	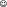	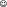
Jayaprakash 2013 (abst)^31^	?	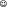	?	?	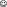	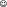	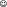
Jayaprakash 2009^32^	?	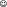	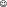	?	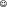	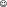	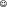
Kammerer 2015^29^	?	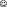	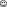	?	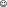	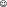	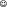
Koch 2011^23^	?	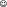	?	?	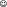	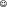	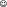
Marzouki 2012^34^	?	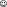	?	?	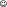	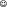	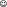
McIntosh^24^ 2009	?	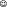	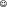	?	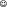	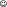	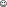
McNamara 2012^25^	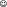	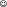	?	?	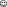	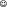	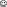
Patel 2011^33^	?	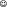	?	?	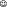	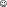	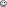
Piazza 2016^17^	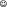	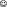	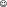	?	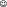	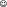	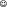

*Note*: (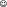) Low risk; (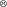) high risk; (?) unclear risk.

### 3.4 Diagnostic performance of COE (summary of results)

Despite wide variation in the types of dental and medical professionals performing the COE and the methodology used for undertaking biopsy across the included studies, meta‐analyses were conducted, where possible, for all relevant outcomes.

#### 3.4.1 Sensitivity and specificity for dysplastic or malignant lesions

The diagnostic performance of COE versus incisional or excisional biopsy for identification of dysplastic or malignant lesions was reported in 18 studies and is summarized in Table 3. The sensitivity varied widely from 25%^22^ to 100%,^21^ as did the specificity from 24%^21^ to 100%.^19^, ^29^

**TABLE 3:** COE versus incisional or excisional biopsy: Sensitivity and specificity for dysplastic/malignant lesions

Author, Year	Description of positive/negative case definition by reference standard test as reported	Dental professional performing index test	Prevalence: Dysplasia or malignant lesions (positive test)	TP	FN	FP	TN	Sens	Spec	PPV	NPV
Studies included in meta‐analysis											
Allegra 2009^20^	Pos: Dysplasia or malignant Neg: Benign	Experienced otolaryngologist	30/45 (67%)	16 [C: 6 CiS: 3 SiD: 3 MoD:2 MiD: 2]	14 [C: 1 CiS: 1 SiD: 3 MoD:3 MiD: 6]	3	12	53%	80%	84%	46%
Bhoopathi 2011^21^	Pos: Dysplasia or malignant Neg: No dysplasia	Oral surgeons	12/148 (8%)	12	0	104	32	100%	24%	10%	100%
Farah 2012^22^	Pos: Dysplasia Neg: No dysplasia	Oral medicine specialists	28/118 (24%)	7	21	16	74	25%	82%	30%	78%
Forman 2015^30^	Pos: Dysplasia, moderate to severe cellular atypia or malignant Neg: Benign	Residents (<5 years' experience) or surgeons (25–30 years' experience)	74/1003 (7.4%)	36	38	18	911	49%	98%	67%	96%
Gillenwater 1998^19^	Pos: Dysplasia or malignant Neg: Normal, no dysplasia	Experienced neck and head surgeon or dental oncologist	17/28 (61%)	13	4	0	11	76%	100%	100%	73%
Hanken 2013^28^	Pos: Dysplasia or premalignant Neg: No lesions	Experienced Oral and Maxillofacial surgeon	54/60 (90%)	41	13 [C: 0 Grading of dysplasia NR]	4	2	76%	33%	91%	13%
Jayaprakash 2013^31^ (abst)	Pos: Dysplasia, carcinoma in situ or malignant Neg: Normal/Benign	NR	184/255 (72%)	99	85	38	33	54%	46%	72%	28%
Jayaprakash 2009^32^	Pos: Dysplasia or malignant Neg: Normal/Benign	Specialist dental oncologist	170/249 (68%)	89 [C: 12 CiS/microinvasive SCC: 5 SeD: 6 MoD: 13 MiD/PA:53]	81 [C: 3 CiS/microinvasive SCC: 5 MoD: 5 MiD/PA:68]	24	55	52%	70%	79%	40%
Kammerer 2015^29^	Pos: Moderate dysplasia (squamous intraepithelial neoplasia >1), malignant Neg: Normal or with inflammatory alterations	NR	10/50 (20%)	9	1 [SCC:0 MoD:1]	0	40	90%	100%	100%	98%
Koch 2011^23^	Pos: Dysplasia or malignant (suspicious for premalignant or malignant lesions) Neg: Benign or no dysplasia (abnormal but innocuous)	Specialist dental oncologist	33/78 (42%)	31	2 [SCC:1 Grading of dysplasia NR]	1	44	94%	98%	97%	96%
Marzouki 2012^34^	Pos: Dysplasia or malignant Neg: Non suspicious	Head and neck oncology staff person	13/33 (40%)	8	5	9	11	62%	55%	47%	69%
McIntosh 2009^24^	Pos: Dysplasia or malignant Neg: Benign	NR	9/50 (18%)	7	2	12	29	78%	71%	37%	94%
McNamara 2012^25^	Pos: Premalignant, carcinoma in situ or malignant Neg: Benign	NR	3/34 (9%)	2 [C:1 SeD: 1]	1 [SeD]	1	30	67%	97%	67%	97%
Patel 2011^33^	Pos: Premalignant or malignant Neg: Benign	Specialists dentists with postgrad qualifications	350/2079 (17%)	292	58	186	1543	83%	89%	61%	96%
		General dental practitioners	32/404 (8%)	28	4	9	363	88%	98%	76%	99%
		All clinicians combined	391/2517 (16%)	327	64	196	1930	84%	91%	63%	97%
Studies not included in meta‐analysis											
Brocklehurst 2015^18^ ^,^ ^a^	Pos: Potentially malignant disorders or malignant Neg: Benign	Primary care dentists	35/90 (39%)	NR	NR	NR	NR	Median 81% (32–100)	Median 73% (32–97)	NR	NR
		Hygienists/therapists	35/90 (39%)	NR	NR	NR	NR	Median 77% (35–100)	Median 69% (42–90)	NR	NR
		Hospital based dentists	35/90 (39%)	NR	NR	NR	NR	Median 90% (81–100)	Median 76% (68–88)	NR	NR
		Dental nurses	35/90 (39%)	NR	NR	NR	NR	Median 68% (48–87)	Median 59% (41–92)	NR	NR
Chainani‐Wu 2015^26^ ^,^ ^b^	Pos: Severe dysplasia, carcinoma in situ or carcinoma Neg: Hyperkeratosis, mild or moderate dysplasia	NR	17/77 (22%)^b^	14 [C: 5 CiS: 9]	3 [C: 1 CiS: 2]	29	31	82%	52%	33%	91%
Epstein 2003^27^ ^,^ ^c^	Pos: Carcinoma or carcinoma in situ Neg: Benign (keratosis, hyperkeratosis, hyperplasia) or dysplasia	NR	30/96 (31%)^c^	12	18	45	21	40%	32%	36%	54%
Piazza 2016^17^ ^,^ ^d^	Pos: Dysplasia or carcinoma Neg: Chronic mucositis, lichen planus without atypia, and keratosis without atypia	NR	87/128 (68%)	44	43	13	28	51%	68%	77%	39%

Abbreviations: Abst, conference abstract; C, carcinoma; CiS, carcinoma in situ; FN, false negative; FP, false positive; MoD, moderate dysplasia; MiD, mild dysplasia; NPV, negative predictive value; PA, parakeratosis with atypia PPV, positive predictive value; SCC, squamous cell carcinoma; Sens, sensitivity; Spec, specificity; SeD, severe dysplasia; TN, true negative; TP, true positive.

^a^ Brocklehurst 2015—Sen and spec reported as median.

^b^ Chainani‐Wu 2015—Data reported for severe dysplasia and cancer only.

^c^ Epstein 2003—Data reported for cancer or CIS only, not dysplasia.

^d^ Piazza 2016—Data reported for COE versus excisional biopsy of the entire lesion under local or general anesthesia regardless of the appearance at COE.

**FIGURE 2:**
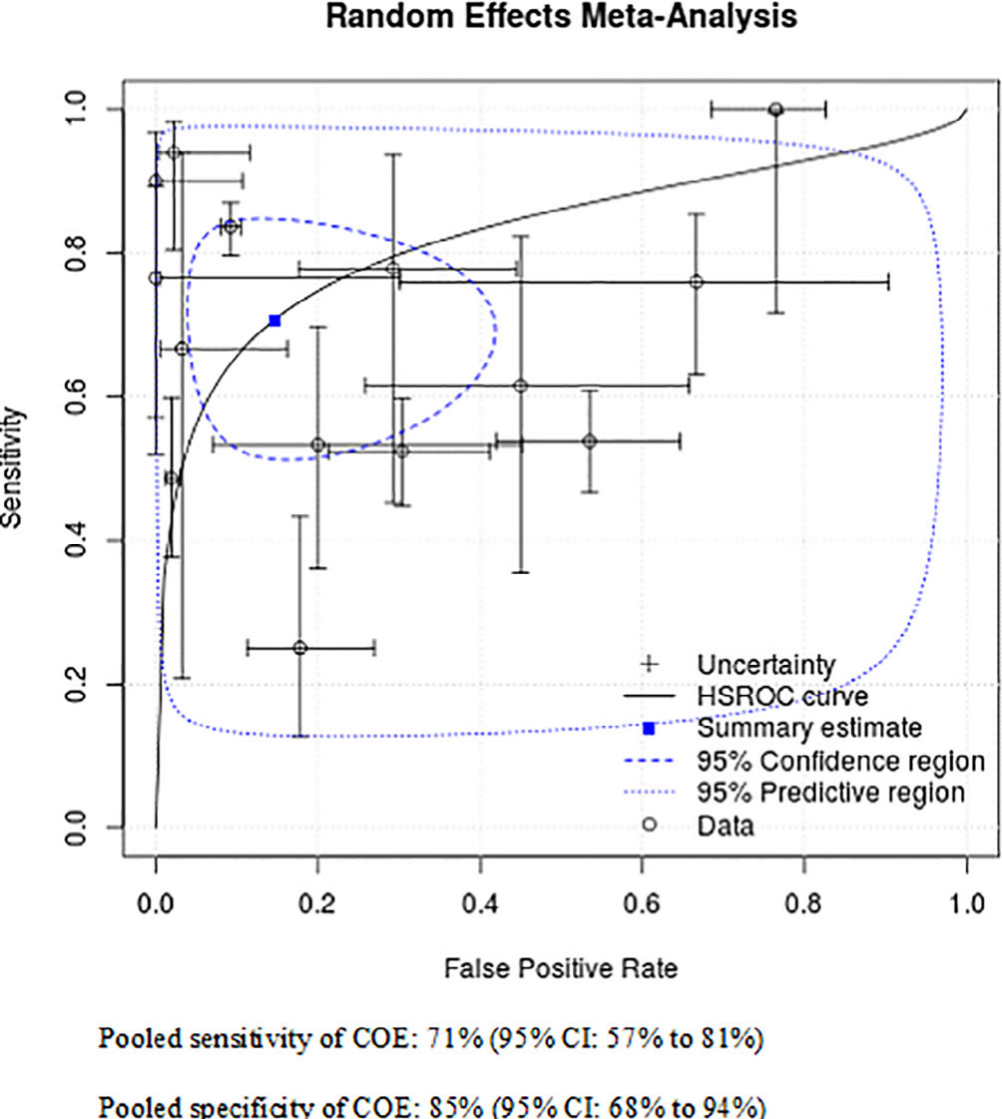
COE versus biopsy: Summary ROC curve for dysplastic and malignant lesions (*N* = 14 studies) [Color figure can be viewed at wileyonlinelibrary.com]

Fourteen^19^, ^20^, ^21^, ^22^, ^23^, ^24^, ^25^, ^28^, ^29^, ^30^, ^31^, ^32^, ^33^, ^34^ studies were included in the meta‐analysis of COE versus incisional biopsy which considered all dysplasia and carcinoma as test‐positive. Four studies were not eligible for meta‐analysis.^17^, ^18^, ^26^, ^27^ Chainani‐Wu et al.^26^ and Epstein et al.^27^ did not consider all grades of dysplasia as test positive, while Piazza et al.^17^ compared COE versus excisional biopsy, and Brocklehurst et al.^18^ did not report appropriate diagnostic performance data. The summary estimate for sensitivity of COE was 71% (95% CI: 57%–81%), the specificity was slightly better at 85% (95% CI: 68%–94%). The false positive rate was 15% (95% CI: 6%–32%) and the false negative rate was 29% (95% CI: 19%–43%). The summary receiver operating characteristic (ROC) curve using a random effects model is presented in Figure 2.

#### 3.4.2 Diagnostic accuracy in identifying different types of lesions

Studies reporting data on the diagnostic performance of s COE versus incisional or excisional biopsy for the identification of different types of lesions, for example, mild dysplasia, moderate dysplasia, severe dysplasia, micro invasive carcinoma, and carcinoma in situ was scarce. Six studies reported albeit limited data on the true positive and/or true negative findings for different types of lesions (see Table 3). In Jayaprakash et al.,^32^ the majority of false negative findings were due to misdiagnosis of mild dysplasia or parakeratosis with atypia 68/81 (84%) and 3/81(4%) related to the missed diagnosis of cancer. Similarly in Allegra et al.,^20^ 6/14 (43%) false negative findings were due to a missed diagnosis of mild dysplasia. The remaining false negative findings consisted of moderate dysplasia (*n* = 3), severe dysplasia (*n* = 3), carcinoma in situ (*n* = 1), and cancer (*n* = 1). While Kammerer et al.^29^ and McNamara et al.^29^ reported only one false negative finding due to moderate dysplasia and severe dysplasia, respectively. Hanken et al.^28^ and Koch et al.^23^ did not report on the grading of dysplasia hence the false negative findings could not be explored further. Lastly, Chainani‐Wu et al.^26^ categorized mild and moderate dysplasia as negative test. The limited and inconsistency in the data reporting precluded any further analyses.

#### 3.4.3 Sensitivity and specificity for malignant lesions only

**FIGURE 3:**
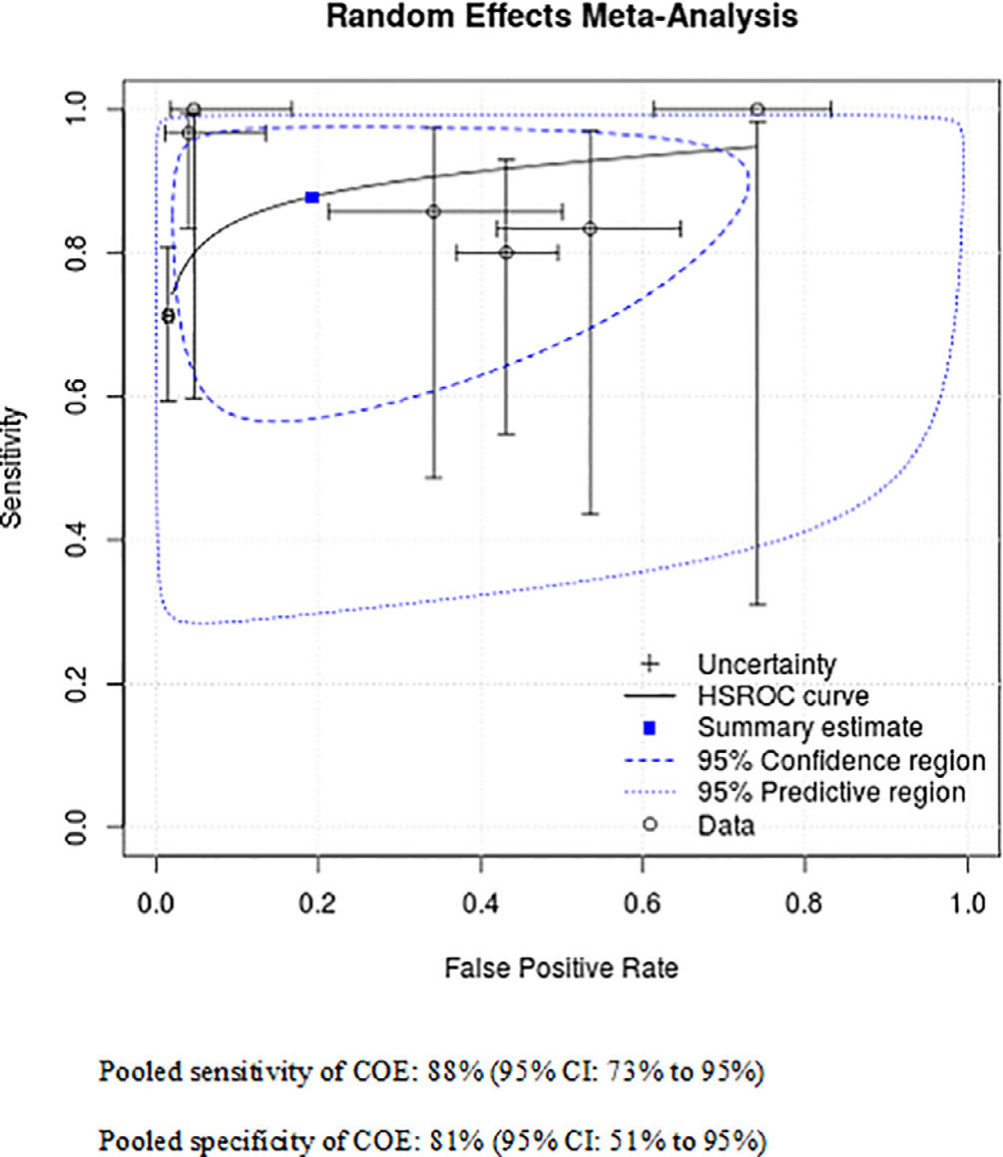
COE versus biopsy: Summary ROC curve for malignant lesions only (*N* = 7 studies) [Color figure can be viewed at wileyonlinelibrary.com]

Seven studies reported data allowing calculation of sensitivity and specificity of COE versus incisional biopsy for identification of malignant‐only lesions (Table 4). The sensitivity varied from 67%^33^ to 100%,^28^, ^29^ and specificity from 26%^28^ to 99%.^33^ A meta‐analysis of the seven studies found overall pooled estimates of 88% (95% CI: 73%–95%) and 81% (95% CI: 51%–95%) for sensitivity and specificity, respectively. The false positive rate was 19% (95% CI: 5.5%–49%) and the false negative rate was 12% (95% CI: 5%–27%). The summary ROC curve is presented in Figure 3.

**TABLE 4:** COE versus incisional or excisional biopsy: Sensitivity and specificity for malignant lesions only

Author, year	Description of positive/negative case definition by reference standard test as reported	Dental professional performing index test	Prevalence of oral invasive cancer	TP	FN	FP	TN	Sens	Spec	PPV	NPV
Allegra 2009^20^	Pos: Carcinoma Neg: Non‐carcinoma	Experienced otolaryngologist	7/45 (16%)	6	1	13	25	86%	66%	32%	96%
Chainani‐Wu 2015^26^	Pos: Carcinoma Neg: Non‐carcinoma	NR	6/77 (8%)	5	1	38	33	83%	46%	12%	97%
Hanken 2013^28^	Pos: Carcinoma Neg: Non‐carcinoma	Experienced oral and maxillofacial surgeon	2/60 (3%)	2	0	43	15	100%	26%	4%	100%
Jayaprakash 2009^32^ ^,^ ^a^	Pos: Invasive SCC and other carcinomas Neg: Non‐carcinoma	Specialist dental oncologist	15/249 (6%)	12	3	101	133	80%	57%	11%	98%
Kammerer 2015^29^	Pos: OSCC Neg: Non‐carcinoma	NR	7/50 (14%)	7	0	2	41	100%	95%	78%	100%
Koch 2011^23^	Pos: SCC Neg: Non‐SCC	Specialist dental oncologist	30/78 (38%)	29	1	2	48	97%	96%	94%	98%
Patel 2011^33^	Pos: Malignant Neg: Nonmalignant	Specialists dentist with registered postgraduate qualifications	58/2079 (2.8%)	42	16	30	1991	72%	99%	58.%	99%
		General dental practitioners	3/404 (0.7%)	2	1	5	396	67%	99%	29%	100%
		All clinicians combined	66/2517 (2.6%)	47	19	36	2415	71%	99%	57%	99%

Abbreviations: FN, false negative; FP, false positive; NPV, negative predictive value; OSCC, oral squamous cell carcinoma; PPV, positive predictive value; SCC, squamous cell carcinoma; Sens, sensitivity; Spec, specificity; TN, true negative; TP, true positive.

^a^ Data calculated for invasive SCC and other carcinomas (one salivary gland carcinoma + two verrucous carcinomas with SSC component).

#### 3.4.4 Diagnostic accuracy of different types of dental professional in identifying dysplastic or malignant lesions

Three studies compared the diagnostic accuracy of different dental professionals performing COE to identify dysplastic or malignant lesions (Table 3). Brocklehurst et al.^18^ compared primary care dentists, hygienists/therapists, hospital‐based dentists, and dental nurses. Sensitivity and specificity were reported as median and range for each dental professional, with a higher sensitivity (median 90%) and specificity (median 76%) being achieved when COE was performed by hospital‐based dentists, followed by primary care dentists (sensitivity 81%, specificity 73%); hygienists/therapists (sensitivity 77%, specificity 69%) and dental nurses (sensitivity 68%, specificity 59%). Patel et al.^33^ also compared diagnostic accuracy between general dental practitioners and specialist dentists (a dentist with registered postgraduate training and qualifications). In contrast to Brocklehurst et al.,^18^ Patel et al.^33^ reported higher sensitivity (88%) and specificity (98%) for general dental practitioners compared with specialist dentists (sensitivity 83%, specificity 89%). Forman et al.^30^ did not report sensitivity or specificity data for each dental professional but reported that the overall accuracy of identifying benign lesions from non‐benign lesions was greater for experienced surgeons with 25–30 years of experience compared with surgeons with less than 5 years' experience (96% vs. 94%, respectively).

#### 3.4.5 Diagnostic accuracy of different types of dental professional in identifying malignant lesions only

Only one study, Patel et al.^33^ assessed this outcome by dental professional (Table 4). The study showed no difference in specificity (99%) between general dental practitioners and specialist dentists with registered postgraduate qualifications. However, a slightly higher sensitivity (72% vs. 67%) was observed with specialist dentists.

## 4 DISCUSSION

### 4.1 Summary of results

In this systematic review, 18 studies were identified that assessed the diagnostic performance of COE versus incisional or excisional biopsy for identification of dysplastic or malignant lesions. Of these, 14 studies were included in a random effects meta‐analysis giving a summary estimate for COE of 71% sensitivity and a slightly better specificity of 85% with a false positive and false negative rate of 15% and 29%, respectively. The diagnostic accuracy of identifying malignant‐only lesions was reported in seven studies, giving a pooled estimate of 88% sensitivity and 81% specificity. The false positive rate was 19% and the false negative rate was 12%. However, it should be noted that due to the multifocal nature of oral premalignant lesions, pathologic analysis of random biopsies often does not provide accurate information of the status of the entire clinically evident lesion hence caution should be applied when looking at the sensitivity and specificity estimates of COE versus incisional or excisional biopsy. In addition, we identified three studies that compared the diagnostic accuracy of different dental professionals performing COE to identify dysplastic or malignant lesions. Varying sensitivity and specify was observed depending on who performed the assessment with higher accuracy observed when performed by a dentist with registered postgraduate training and qualifications. Unfortunately, due to the limited number of studies and evidence, this did not allow for a definitive conclusion to be made regarding the most appropriate dental or medical professional to conduct COE.

### 4.2 Interpretation of results and comparison to the existing literature

The findings of our review showed wide variation in sensitivity and specificity between studies, though the pooled diagnostic accuracy of COE is comparable with detection of other cancers (e.g., colon–rectum, cervix, or breast cancer) by clinical inspection or other noninvasive approaches.^35^, ^36^, ^37^ While there are many screening programs for OCC or OPMDs, a systematic review by Warnakulasuriya et al.^38^ found that visual screening by dentists in primary care or in extended health care facilities can accurately identify OCC and/or OPMDs with reliability when using established guidelines. Walsh et al.^39^ reported that COE was better at correctly classifying the absence of OPMDs or oral cavity cancer in disease‐free individuals than classifying the presence in diseased individuals. The number of false negative and false positive findings are a potential concern. In the case of the false negative outcomes, where the disease is not detected and left untreated, the cancer may reach an advanced stage which could lead to late diagnosis and poorer prognosis. This would be especially detrimental in the case where a high‐grade dysplastic lesion or a malignant lesion was left undetected in contrast to a less invasive lesion such as a mild dysplastic lesion with a very low risk of cancer development. Thus further exploration of the number of false negative outcomes across malignant and premalignant lesions was undertaken. Unfortunately only a limited number of studies reported the false negative outcomes across the different types of lesions (see Tables 3 and 4), precluding further analysis. The major surgical treatment needed for the advanced stage disease could result in disfigurement, social isolation, increased levels of morbidity, and infrequently, death.^40^, ^41^, ^42^ There is a consensus view that early detection, diagnosis and treatment of OCC can significantly enhance survival rates and reduce morbidity.^43^ Silverman et al.^44^ researched the relationship between delay in diagnosis, stage, and mortality and state that survival rates would increase by 80% if malignancies were identified and treated earlier. In addition, patients with false positive findings will be undergoing unnecessary biopsy, causing unnecessary anxiety, worry and cost and impacting the patients' quality of life. Commonly observed diagnoses among false positive findings within and across the included studies were not reported. However, Chainani Wu et al.^26^ reported that the clinical sign of speckled appearance had a high sensitivity for the detection of carcinoma in situ or carcinoma though the specificity for speckled appearance was low. Meanwhile, Forman et al.^30^ hypothesized that there were certain common oral lesions that were associated with a high degree of clinical diagnostic accuracy. Fibromas (99.2%), mucoceles (97.2%), pyogenic granulomas (96.8%), and squamous papillomas (96.3%) showed a high level of concordance. Traumatic ulcerations were associated with a clinical impression accuracy rate of 83.6%. In addition, the authors reported that older patients and patients who received radiation therapy were most likely to be misdiagnosed clinically and men were 1.5 times more likely to be misdiagnosed.

### 4.3 Strengths and limitations

The strength of our review lies in the robust systematic reviewing methodology used, including the comprehensive and reproducible search strategy. There are a number of limitations to our review. Primarily, there was significant heterogeneity in all aspects of study design across the studies, including patient characteristics, previous history of cancer, prevalence of dysplastic and malignant lesions among the study sample, the range of clinical lesions, the types and expertise of the dental and medical professional performing the COE and the methodology used for undertaking biopsy across the included studies. In addition, there was lack of consistency and reporting across the included studies in the clinical criteria used to define levels of “suspicion” for making a clinical assessment of benign versus malignant. Another potential limitation of our review could be the inclusion of retrospective studies, especially the inclusion of Patel et al.,^33^ which was a large study with a cohort of 3067 participants. However a sensitivity analysis with the exclusion of this study made minimal impact on the sensitivity (69% vs. 71%) and specificity (85% vs. 85%) results, see Appendix S3. Furthermore, the sensitivity and specificity of COE in this review might be overestimated as the majority of the included studies have been conducted by specialists with an interest in OCC with extensive experience, unlike a general dental or medical clinician, hence limiting the applicability of the review findings to general practice. It is important to note that these limitations are principally sourced in the evidence base, rather than the methods used to interrogate, and evaluate it.

### 4.4 Implications for policy, practice, and future research

Our review highlights the need to identify ways to improve the accuracy of detecting OCC and OPMDs at an early stage of the disease. Improvements in making a diagnosis may be sought through continuous training in accurate interpretation of diagnostic approaches, care during examination, building experience, expertise, and confidence of dental or medical professionals in detecting OCC or dysplasia, increasing patients' awareness and acceptance of the disease and developing reliable adjunctive tools to improve findings and accuracy of COE. Currently, research is ongoing into the effectiveness of the use of adjunctive aids such as toluidine blue, chemiluminescence, loss of tissue autofluorescence and other aids.^22^, ^24^, ^43^, ^45^, ^46^ However, as yet there is insufficient evidence to justify their use as adjuncts to COE.^43^, ^47^, ^48^ While there is a growing body of research investigating detection technologies and new therapies, psychosocial research into how these new developments may be accepted and utilized is also required.^43^ A review by Ford et al.^43^ looked at a theoretical model^49^ to explore behavioral influences on the early detection of OCC. The model comprised four time intervals (appraisal; help seeking; diagnostic; and pretreatment) that made up the total time between the appearance of signs or symptoms of a cancer and the commencement of treatment. The authors^43^ proposed that unless future theory‐based studies target these aspects of OCC, and consider the structural and psychosocial parameters that surround it, then efforts to improve its timely detection will have limited effectiveness. In addition they also suggested addressing health inequity at a government policy level and focusing on improved access and affordability since low socioeconomic status is a risk factor for OCC.^43^ The most important limitation identified in our review was the inconsistency in the clinical assessment of negative and positive test and differentiation between the level of suspicion, that is, low or high risk lesion. Therefore, further research in developing standardized assessment criteria is needed as well as potentially exploring the use of artificial intelligence approaches.

## 5 CONCLUSIONS

The pooled diagnostic accuracy of COE versus incisional or excisional biopsy for identifying dysplastic and/or malignant lesions is 71% sensitivity and 85% specificity, with a slightly better sensitivity of 88% and specificity of 81% for identifying malignant lesions only. The evidence on diagnostic accuracy for different types of dental or medical professional in identifying dysplastic or malignant lesions was inconclusive due to the limited number of studies identified. This review highlights the need to improve the diagnostic accuracy of detecting malignant and/or potentially malignant oral lesions at an early stage of the disease in order to improve prognosis and outcomes. In addition, further well‐designed studies are needed to compare the accuracy of different members of the dental and medical team in detecting malignant and nonmalignant oral lesions.

## CONFLICT OF INTEREST

The authors declare that they have no conflict of interest.

## AUTHOR CONTRIBUTIONS

All authors contributed to the study conception and design. Material preparation, data collection and analysis were performed by Munira Essat, Katy Cooper, and Mark Clowes. The first draft of the manuscript was written by Munira Essat and all authors commented on previous versions of the manuscript. All authors read and approved the final manuscript.

## Supporting information


**Appendix S1**: Example search strategy
**Appendix S2**: Eligibility criteria for study selection
**Appendix S3**: COE versus biopsy: Summary ROC curve for dysplastic and malignant lesions without Patel et al. 33 (*N* = 13 studies)

## Data Availability

The data that supports the findings of this study are available in the supplementary material of this article.
