# Supplementary material for: Diagnostic accuracy of conventional oral examination for detecting oral cavity cancer and potentially malignant disorders in patients with clinically evident oral lesions: Systematic review and meta‐analysis
Source: Head Neck. 2022 Jan 29;44(4):998–1013. doi: 10.1002/hed.26992 (PMC9306506; doi:10.1002/hed.26992)
Supplement: Supplementary file 1 — Appendix S1: Example search strategy Appendix S2: Eligibility criteria for study selection Appendix S3: COE versus biopsy: Summary ROC curve for dysplastic and malignant lesions without Patel et al. 33 (N = 13 studies) [file HED-44-998-s001.pdf]

Appendices

Appendix 1: Example search strategy

This strategy was used for MEDLINE and adapted for the other databases searched (see section 2 of main report for details).

Database: Ovid MEDLINE(R) and Epub Ahead of Print, In-Process & Other Non-Indexed Citations, Daily and Versions(R)

Search Strategy:

----

1 exp Mouth Neoplasms/

2 ((oral or dent* or mouth) adj3 (cancer* or precancer* or malignan* or premalignan* or dysplasia)).mp. [mp=title, abstract, original title, name of substance word, subject heading word, floating sub-heading word, keyword heading word, organism supplementary concept word, protocol supplementary concept word, rare disease supplementary concept word, unique identifier, synonyms]

3 1 or 2

4 specificity.tw.*

5 3 and 4

6 biops*.mp.

7 (discordan* or concordan* or reliab* or concurren* or accura* or unreliab*).mp.

8 3 and 6 and 7

9 5 or 8

**McMaster University high precision filter for identifying diagnostic studies*
(https://hiru.mcmaster.ca/hiru/HIRU_Hedges_MEDLINE_Strategies.aspx)

Appendix 2: Eligibility criteria for study selection

	Inclusion	Exclusion
**Study design**	• Diagnostic accuracy study (cohort study with index test and reference standard)	• Other study designs (not diagnostic accuracy)
**Population**	• Patients with oral lesion or oral potentially malignant disorders	• Healthy adults with no visible lesion<br>• Other cancers: see below
**Condition being diagnosed**	1) Oral potentially malignant disorders i.e. dysplastic and malignant lesions (including oral cancer, carcinoma in situ and all types of dysplasia: mild, moderate and severe)<br><br>2) Malignant lesions alone	• Metastases to lymph nodes<br>• Neck cancer / lesions<br>• Thyroid cancer<br>• Laryngeal cancer<br>• Pharyngeal and nasopharyngeal cancer<br>• Salivary gland & parotid cancer<br>• Lip cancer
**Index test**	• Conventional oral examination (visual inspection)	• Other non-invasive adjunctive visual tools e.g. toluidine blue, Vizilite, auto fluorescence spectroscopy etc.<br>• SNB / SLNB<br>• PET / CT / MRI / ultrasound<br>• DNA / RNA / protein markers<br>• FNAC or CNB for salivary gland / parotid tumours<br>• Methods of processing or assessing biopsied tissue, e.g. staining, freezing, cytology, pathologist concordance
**Reference standard**	• Biopsy<br>• Full excision of lesion	• Visual inspection by another clinician<br>• Other reference standards
**Outcome measures**	• Sensitivity & specificity<br>• Other diagnostic measures e.g. positive and negative predictive value<br>• Concordance<br>• Accuracy for different types of dental professional	
**Dates of publication**	• 1990 onwards	• Studies published before 1990
**Country of study**	• Developed countries e.g. UK, Western Europe, Australia, North America, Canada, Japan, South Korea	• Developing countries and countries with different health system (e.g. Eastern Europe and Middle East e.g. Poland, Romania, Israel)
**Language**	• English, or sufficient English to extract data	• Insufficient English to extract data

CNB, core needle biopsy; COE, conventional oral examination; CT, computed tomography; DNA, deoxyribonucleic acid; FNAC, fine needle aspiration cytology; MRI, magnetic resonance imaging; PET, positron emission tomography; RNA, ribonucleic acid; SNB / SLNB, sentinel (lymph) node biopsy; UK, United Kingdom

Appendix 3: COE vs. biopsy: Summary ROC curve for dysplastic and malignant lesions without Patel et al[33] (N=13 studies)

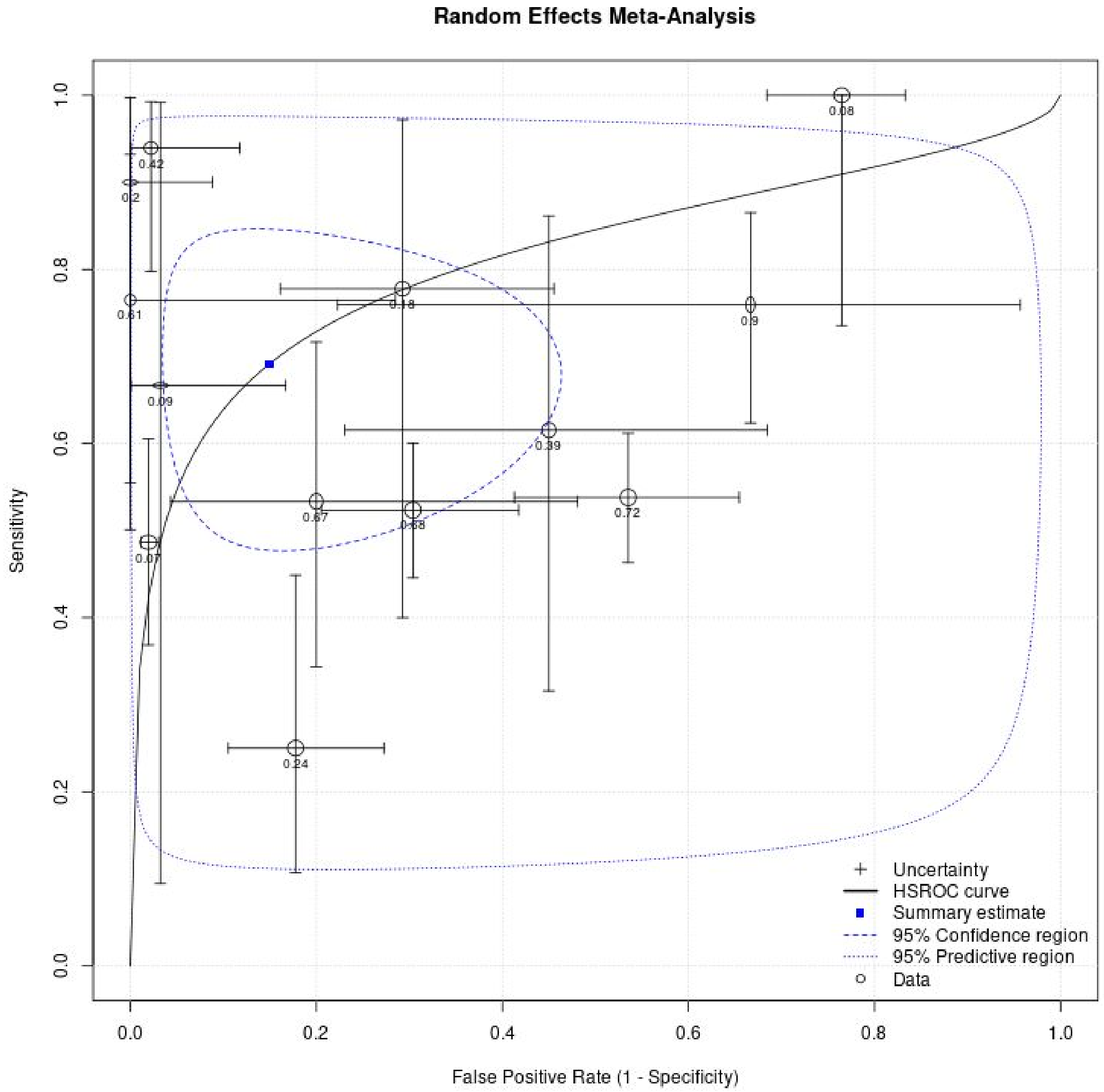

Pooled sensitivity of COE: 70% (95% CI: 55% to 81%)

Pooled specificity of COE: 85% (95% CI: 65% to 95%)
